# Salt-Induced Changes in the Phenolic Content of Melon F2 Offspring Sprouts Obtained from Fruit Deseeding

**DOI:** 10.3390/foods14132242

**Published:** 2025-06-25

**Authors:** Angelica Galieni, Beatrice Falcinelli, Fabio Stagnari, Federico Fanti, Eleonora Oliva, Paolo Benincasa

**Affiliations:** 1 Research Centre for Vegetable and Ornamental Crops, Council for Agricultural Research and Economics [CREA-OF], 63077 Monsampolo del Tronto, Italy; angelica.galieni@crea.gov.it; 2 Department of Agricultural, Food and Environmental Sciences, University of Perugia, 06121 Perugia, Italy; paolo.benincasa@unipg.it; 3 Department of Bioscience and Technology for Agriculture Food and Environment, Campus Universitario di Coste Sant’Agostino, University of Teramo, 64100 Teramo, Italy; fstagnari@unite.it (F.S.); ffanti@unite.it (F.F.); eoliva@unite.it (E.O.)

**Keywords:** germination, polyphenols, flavonoids, salinity, elicitation, food supplement

## Abstract

This study investigated the phytochemical content of melon sprouts obtained from by-product seeds of fruit processing and the elicitation effect obtained by the application of salinity to the growing substrate. Seeds from two melon Cultivars (Thales and SV9424ML) were sprouted at 0, 12.5, 25, and 50 mM NaCl concentrations (Salt). Due to intra-lot seed variability in germination speed, sprouts were harvested at 1 and 2 weeks after sowing (WAS), included as an experimental factor (Harvest), collecting, at each harvest, only those that had reached the ready-to-eat stage. Seed germination, shoot and root lengths, fresh and dry weights, and their content in phenolic compounds were determined. Cultivar, Harvest, and Cultivar × Harvest interaction affected sprout phenolic compound content more than Salt. In general, Thales exhibited a significantly greater phenolic compound content (+67.9%, on average). Harvest influenced phytochemicals, with sprouts at 2WAS exhibiting lower flavonoid and hydroxybenzoic acid levels (−31.3% and −73.0%, respectively), yet higher hydroxycinnamic acid content (+298.6%). This was a consequence of variations in *p*-coumaric and ferulic acids at 2WAS and in flavonoids at 1WAS. Moreover, Salt had an appreciable effect only on Thales, at moderate levels (25 mM NaCl). Our results suggest that the sprouting of by-product seeds of vegetables should be finely modulated based on the seed intra-lot variability in germination speed and on cultivar responsiveness to salinity for phytochemical elicitation.

## 1. Introduction

Sprouts are gaining popularity as ready-to-eat micro-scale vegetables, renowned for their appealing taste and rich antioxidant content [1,2,3]. Beyond their direct consumption, sprouts are increasingly valued for their potential to extract and purify phytochemicals for use in food supplements and pharmaceuticals [4,5]. The choice of whether to destine sprouts to direct consumption or phytochemical extraction will also depend on taste quality traits and digestibility, which need to be considered [6]. Commonly, plant families used for sprouting include *Poaceae*, *Brassicaceae*, and *Fabaceae*, with growing interest in species that offer vivid colors, intense aromas, pleasant textures, and diverse flavors [3]. Research opportunities are being expanded to include underutilized species such as wild relatives, ancestors, and neglected or local accessions of cultivated species and fruit tree species. Notably, seeds from fruit species, often by-products of the juice and jam industries, are rich in phytochemicals [6,7]. For instance, in previous studies, we explored sprouts from pomegranate, olive, and *Citrus* species seeds, all by-products of the food industry (see the literature cited within [6]).

The potential use of by-products from vegetable species may offer a promising approach though it remains largely unexplored. In this context, melon (*Cucumis melo* L.)—a widely consumed and economically important fruit—produces considerable by-products from both fresh consumption and industrial processing, with seeds comprising up to 7% of the fruit’s waste weight [8], making them a potential alternative raw material for sprouting. However, for sprout production, it must be considered that the composition and germination performance of melon seeds as well as seedling growth traits exhibit significant variability among genotypes. Moreover, germination parameters can vary within and among plants from which seeds are harvested, influenced by factors such as fruit development timing, fruit position on the plant, harvest timing, and post-harvest storage conditions [9,10]. This variability could be even more pronounced considering the segregation among F2 offspring (seed by-products), as many cultivated varieties are F1 hybrids.

The synthesis of bioactive compounds in plants is regulated by secondary metabolism and influenced by genetic, environmental, and agronomic factors. In the case of phenylpropanoids, the chemical reactions run through the shikimate pathway, which begins with the biosynthesis of 3-deoxy-D-arabinoheptulosonic acid 7-phosphate (DAHP) by the corresponding synthase (DAHPS). DAHP, the first intermediate of the pathway, is converted to chorismite after sic metabolic steps catalyzed by five enzymes. Chorismate is the substrate of at least five other pathways leading to the primary metabolites, such as phenylalanine, which in turn is the primary substrate for phenylpropanoids synthesis, through the action of phenylalanine ammonia-lyase (PAL) [11].

Elicitation techniques, induce oxidative stress and an excess of reactive oxygen species, activating plant responses that enhance phenolic content through increased phenylalanine ammonia-lyase activity [6].

Elicitors are broadly classified into abiotic and biotic types. Abiotic elicitors include physical or chemical factors such as salinity, heavy metals, UV light, and phytohormones (e.g., jasmonic acid, salicylic acid); biotic elicitors derive from biological sources and comprise compounds like chitin, cellulose, microbial extracts, and polysaccharides [12,13,14]. Their controlled application allows for targeted enhancement of phytochemical profiles in sprouts and other plant tissues [6,12].

To this purpose, salt application during sprouting is a widely used abiotic elicitor, representing an accessible and cost-effective method to trigger plant defense mechanisms [6]. Recent studies have shown that low to moderate salt concentrations during germination boost polyphenols and flavonoids in *Lepidium sativum* sprouts [15] and increase phenolic compound levels in durum wheat seedlings [16]. Conversely, saline-alkali stress has been found to reduce flavonoids and phenolic acids in quinoa sprouts [17]. Even in rapeseed sprouts, salt-induced phytochemical changes were linked to both somatic and transgenerational stress memories—with the latter reflecting salt exposure experienced by the parent plants of the offspring seeds [18]. On the other hand, salinity impairs germination by reducing water uptake due to low osmotic potential and by causing ion toxicity (Na^+^, Cl^−^), which disrupts metabolic and hormonal balances essential for seed activation [19,20]. One key finding is that the effect of salt depends on (i) genotype tolerance—including species, botanical varieties, and cultivars—and (ii) salt concentration. Therefore, results based on a few genotypes should be validated across a broader range and varying salt concentrations. While higher stress levels may enhance phytochemical production, they can also inhibit sprout growth, making it essential to finely tune elicitor doses to maximize “phytochemical yield” [21]. Indeed, salt can impair germination and seedling growth parameters [22], as observed in pumpkins, where increasing salinity reduces germination percentages and seedling vigor index and increases mean germination time and ion leakage [23].

Building on these insights, this study aimed to investigate germination performances and phenolic compound accumulation in sprouts obtained from seeds of two distinct melon (*Cucumis melo* L. var. *reticulatus*) cultivars, using different NaCl solution concentrations in the sprouting media. To the best of our knowledge, this is the first study to apply salt as an elicitor during the sprouting of melon seeds and, more broadly, in the production of melon sprouts. Moreover, given the varying germination speed within the same seed batch for each cultivar, we also evaluated the impact of sprout harvest timing to produce ready-to-eat sprouts with fully expanded cotyledons, resulting in two distinct harvest times, differing by about one week.

## 2. Materials and Methods

### 2.1. Seed Materials

Seeds were obtained from two melon HF1 Cultivars, Thales (Syngenta^®^ Sementi Orticole Italia, Syngenta Italia S.p.A., Milan, Italy) and SV9424ML (Seminis, Bayer Vegetables Italia, Bayer Group; Milan, Italy). Commercially mature fruits had been harvested on 28 July 2021, from an open-cultivated melon crop at the Top Melon farm (Top Melon s.r.l., Pantalla, Perugia, Italy). The crop was treated according to the standard agronomic practices used in that area in terms of mulching, nutrient fertilization, irrigation, and protection against pests and diseases. Low tunnels along the furrow were used to limit pollinator circulation until blooming, ensuring more synchronized pollination of pistillate flowers and thus more uniform fruit ripening. Fruits were manually processed to separate seeds, which were then washed and rinsed with tap water to eliminate pulp residues, followed by a final wash with distilled water. A preliminary germination test—performed with two replicates of 100 seeds per Cultivar by laying seeds over Whatman paper wetted with distilled water—revealed a certain variability in the germination speed within each seed lot. Thus, two harvest dates (Harvest)—spaced about one week apart to ensure that only sprouts at the ready-to-eat stage (with fully expanded cotyledons) were harvested on each date—were included as an experimental factor in the sprouting experiment (see Section 2.2).

### 2.2. Sprouting and Experiment Description

Seeds required for any experimental replicate were randomly selected from the obtained bulk seed lot for each Cultivar. Seeds were incubated in plastic trays containing 0, 12.5, 25, and 50 mM of NaCl solutions [Salt treatments (0_mM, 12.5_mM, 25_mM, and 50_mM, respectively)] according to a completely randomized block design with four replicates (trays). The trays were covered by a drilled top to maintain air circulation while preventing dehydration. Distilled water was periodically added to trays to restore initial tray weight, assuming that weight loss was mainly due to water evaporation, approximately keeping the initial NaCl concentration of each treatment. The trays were incubated in a growth chamber at 20 °C in a light–dark regime of 12:12 h. As stated in Section 2.1, to account for intra-seed-lot variability in germination speed, only sprouts that had reached the ready-to-eat stage (fully expanded cotyledons) were sampled for each Harvest. Moreover, since increasing salinity slowed seedling growth, we observed a shift in the time interval needed to reach the ready-to-eat stage among Salt treatments. Thus, for each Cultivar, sprouts of 0_mM and 12.5_mM treatments were harvested 6 days after sowing (DAS) and 14 DAS, while sprouts of 25_mM were harvested at 7 and 15 DAS and those of 50_mM were harvested at 7 and 16 DAS. Hereafter, in the text, the two Harvests will be referred to as 1 and 2 weeks after sowing (WAS), respectively. Fresh and dry weights were measured on a subsample of 20 sprouts per replicate, and the dry matter content (DM, %) was calculated; sprouts were also characterized for their shoot and root lengths (SL and RL, respectively). The remaining sampled sprouts of each replicate were lyophilized, finely homogenized, and stored at −20 °C until the analysis.

### 2.3. Chemicals

All chemicals used were of analytical reagent grade, including protocatechuic acid (ProtA), vanillic acid (VanA), syringic acid (SyrA), caffeic acid (CafA), *p*-coumaric acid (*p*-CouA), ferulic acid (FerA), *trans*-cinnamic acid (*trans*-CinA), apigenin (Api), luteolin (Lut), diosmetin (Dios), orientin (Ori), and naringenin (Nar), all sourced from Sigma-Aldrich (St Louis, MO, USA). Stock solutions of phenolic compounds were prepared in methanol at a concentration of 1.0 × 10^−2^ mol L^−1^ and stored at −20 °C in the dark.

### 2.4. UPLC-ESI-MS/MS Analysis of Phenolic Compounds

The quantitative analysis of phenolic compounds was performed by an Acquity H-Class chromatographic system (Waters, Milford, MA, USA) connected to a Qtrap4500 mass spectrometer (Sciex, Toronto, ON, Canada), following the methodology described by Oliva et al. [24], with minor modifications. Briefly, freeze-dried samples were ground using liquid nitrogen. Approximately 0.1 g of each sample was measured and extracted with 1 mL of a MeOH:H_2_O solution (70:30 *v*:*v*) using ultrasonic-assisted extraction (UAE) for 30 min at room temperature, followed by centrifugation at 11,200 rcf for 10 min at 4 °C. The supernatant was collected, and the pellet was re-extracted under the same conditions. The combined extracts were dried using a SpeedVac Vacuum Concentrator system (Thermo Fischer, Waltham, MA, USA) and the pellet was resuspended with 1 mL of phosphate buffer (50 mM) H_2_O at pH 3:MeOH (90:10 *v*:*v*) for the clean-up phase. The purification step was performed using Solid Phase Extraction (SPE) with Strata XL cartridge (330 mg, 1 mL) from Phenomenex (Torrance, CA, USA), followed by analysis using UPLC-ESI-MS/MS in Multiple Reaction Monitoring (MRM) acquisition modes operating in negative ionization. Analytes were separated by an ACE Excel 2 C18-PFP 2.0 µm (100 × 2.1 mm). For the chromatographic run, H_2_O with 1% of acetic acid and ACN were used for phase A and phase B, respectively. For mass spectrometry acquisition, all phenolic compounds were detected in negative ionization with a capillary voltage of −4500, nebulizer gas (air) at 40 psi and turbo gas (nitrogen) at 40 psi and 200 °C. Data acquisition and processing were conducted using Analyst 1.7.3 software and quantification with Multiquant 3.0.3 software, both from Sciex (Figure S1).

### 2.5. Statistical Analysis

Data were analyzed by three-way ANOVA, and the effects of melon Cultivar, salt level (Salt), and harvest date (Harvest), as well as their interactions (Cultivar × Salt, Cultivar × Harvest, Salt × Harvest, and Cultivar × Salt × Harvest) were tested. ANOVA assumptions were verified through graphical methods. When ANOVA revealed significant differences, means separation was performed through Fisher’s least significant difference test (LSD) at *p* < 0.05. The R statistical environment was used to analyze data [25].

The detected phenolic compounds were analyzed both individually and by grouping them into major chemical classes, as follows: (i) total phenolic compounds (sum of phenolic acids and flavonoids); (ii) total flavonoids (sum of apigenin, luteolin, diosmetin, orientin, and naringenin); (iii) total hydroxybenzoic acids (sum of protocatechuic acid, vanillic acid, and syringic acid); and (iv) total hydroxycinnamic acids (sum of caffeic acid, *p*-coumaric acid, ferulic acid, and *trans*-cinnamic acid).

## 3. Results

### 3.1. Growth Performances of Melon Sprouts

**Figure 1:**
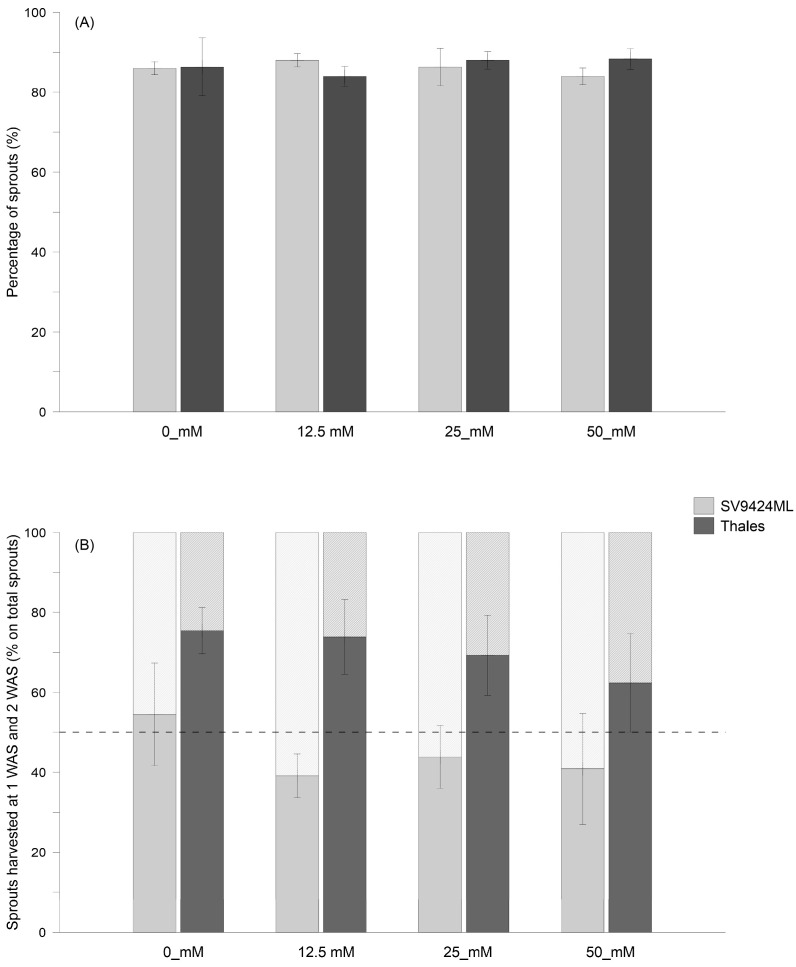
(**A**) Total percentage of sprouts obtained from seeds of two melon Cultivars (SV9424ML and Thales) harvested at 1 and 2 weeks after sowing (WAS) and subjected to different Salt levels during sprouting (0_mM, 12.5_mM, 25_mM, and 50 mM, respectively). In (**B**), the contribution of each harvest date—expressed as the percentage of sprouts harvested at 1 WAS (solid-filled charts) and 2 WAS (forward-slash-filled charts)—is reported.

The overall percentages (1 plus 2 WAS) of sprouts harvested for each Cultivar and salt level are reported in Figure 1A, while Figure 1B shows the contribution percentage of the sprouts harvested at 1 and 2 WAS on the total of the harvested melon sprouts for each Cultivar. Different percentages of sprouts harvested at 1 and 2 WAS depended principally on differences in germination speed within the same seed lot rather than on differences in growth rates caused by different salinity levels.

More specifically, the Cultivar and Salt did not influence the total amount of harvested sprouts (Figure 1A); however, the percentage of sprouts harvested at 1 and 2 WAS sharply varied between SV9424ML and Thales (Figure 1B). On average across Salt treatments, Thales produced more ready-to-eat sprouts than SV9424ML at 1 WAS (60.8% vs. 38.5% of the total amount of harvested sprouts, respectively). On the other hand, SV9424ML showed higher values at 2 WAS (25.8% vs. 47.6% of the total amount of harvested sprouts, averaged across Salt treatments, for Thales and SV9424ML, respectively). Also, the Salt level enhanced the percentage—on total amount—of sprouts harvested at 2 WAS regardless of the cultivar (Figure 1B). The percentages of sprouts harvested at 2 WAS ranged from 39.3% at 0_mM to 53.3% at 12.5_mM for SV4224SL and from 22.3% at 0_mM to 32.7% at 50_mM for Thales.

The variations observed in RL were influenced by the effects of Cultivar, Salt, and Harvest (Table 1).

In general, higher RL values were observed in Thales, with an overall +18.9% compared to SV9424. RL was also significantly higher under 0_mM (106.9 cm) compared to 50_mM, when averaged across Cultivars and Harvests. Additionally, seedlings at 2 WAS showed greater RL (105.5 cm) than those harvested at 1 WAS (95.0 cm), on average. Interestingly, in Thales, a 25_mM salt solution induced higher RL values at both 1 WAS and 2 WAS; this clear trend was not observed for SV9424ML (Table 1). SL was also influenced by Cultivar, salt concentration, and harvest time. A significant reduction was observed at 50_mM, regardless of Cultivars and Harvests (Table 1). This variation was primarily due to the decrease in SL values in sprouts harvested at 1 WAS (−33% for 50_mM compared to 0_mM, averaged across Cultivars) rather than at 2 WAS (no significant variation among Salts). So, the effect of Cultivar was particularly evident at 1 WAS, where at 50_mM, reductions of 43% and 23% were observed for SV9424ML and Thales, respectively (Table 1). These differences resulted in changes in the SL/RL ratios (Table 1). Among the most interesting effects, sprouts harvested at 1 WAS showed a significant reduction (at *p* < 0.01) in SL/RL value starting from 25_mM for Thales and 50_mM for SV9424ML (Table 1).

Thales produced, on average, sprouts characterized by higher FW values than SV9424ML (233.0 vs. 207.5 mg sprout^−1^). Moreover, low salinity levels in the sprouting media seemed to increase FW values with the best-performing salinity level varying with Harvests, as the result of different germination speeds (25_mM at 1 WAS and 12.5_mM at 2 WAS, regardless of Cultivars) (Table 1). The DM concentration values were higher in Thales than SV9424ML (6.37% vs. 5.95%) and were significantly influenced by the salinity level, with generally higher values at 50_mM, regardless of Cultivars and Harvests. For SV9424ML we observed a 6.45% DM at 1 WAS vs. 5.45% DM at 2 WAS, averaged across Salts (Table 1).

### 3.2. Phytochemical Profiles of Melon Sprouts

#### 3.2.1. Total Phenolic Compounds

**Figure 2:**
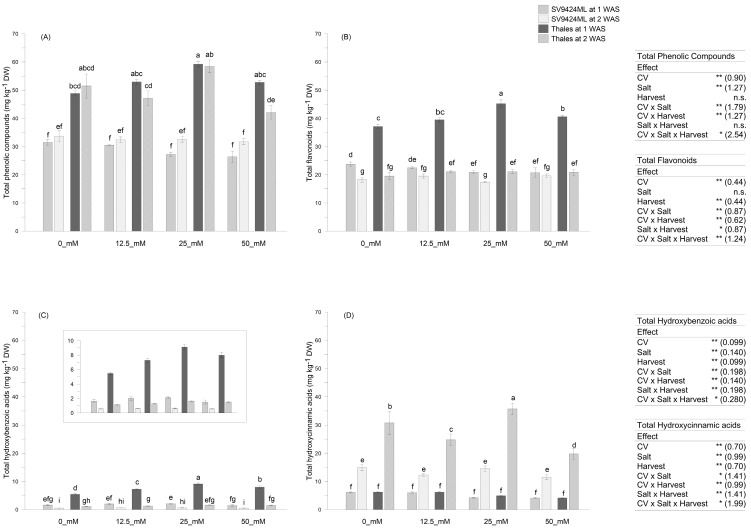
Phenolic compounds (mg kg^−1^ dry weight, DW) as observed in sprouts of two melon Cultivars (SV9424MLand Thales) subjected to different Salt treatments during sprouting (0_mM, 12.5_mM, 25_mM, and 50 mM). Sprouts were harvested (Harvest) at 1 week after sowing, WAS (solid-filled bars), and at 2 WAS (slash-filled bars)—were sampled. (**A**) Total phenolic compounds; (**B**) total flavonoids; (**C**) total hydroxybenzoic acids; (**D**) total hydroxycinnamic acid. Data represent means ± standard errors (*n* = 4). In the boxes, the results of the three-way ANOVA (degrees of freedoms: Cultivar, 1; Salt, 3; Harvest, 1; Cultivar × Salt, 3; Cultivar × Harvest, 1; Cultivar × Salt × Harvest, 3; residues, 48); n.s. not significant; * *p* < 0.05; ** *p* < 0.01; numbers on the brackets represent the standard errors of the differences between means (s.e.d.). Lowercase letters indicate significant differences in the Cultivar × Salt × Harvest interaction at *p* < 0.05 (Fisher’s Least Significant Difference, LSD).

**Figure 3:**
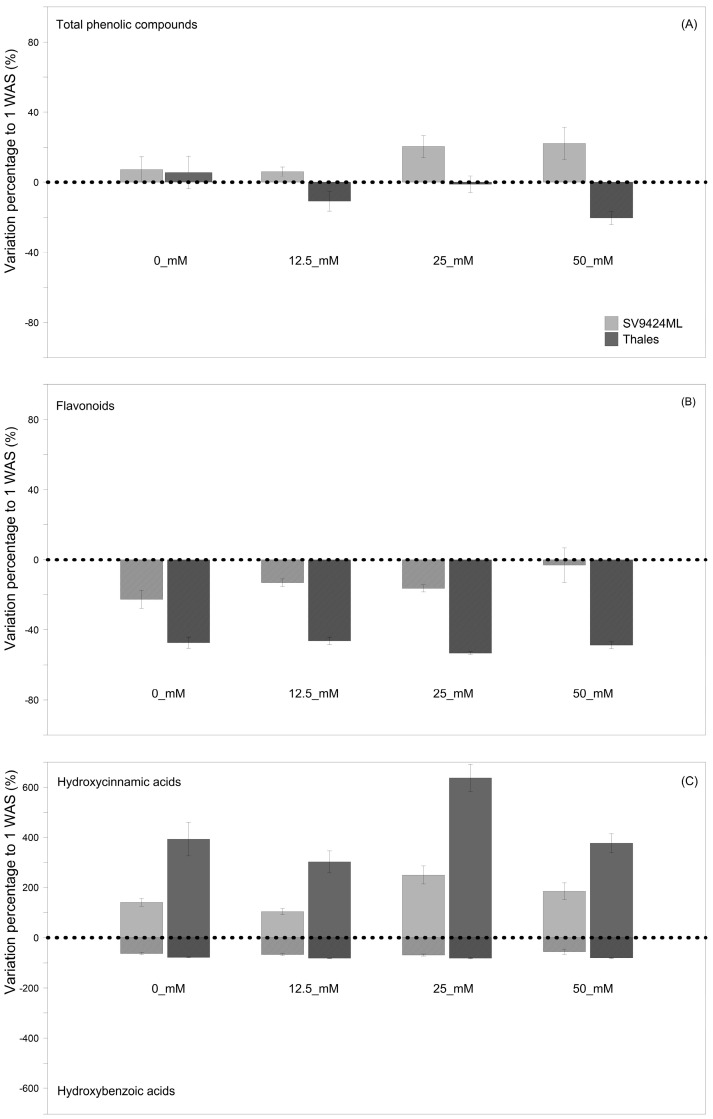
Variation percentage (%) of the single classes of the investigated phenolic compounds as recorded at the second harvest date (2 weeks after sowing, WAS) to the first one (1 WAS) values (dotted horizontal lines). Figures concern the following: (**A**) total phenolic compounds; (**B**) total flavonoids; (**C**) total hydroxybenzoic acids (lower half) and total hydroxycinnamic acids (upper half). Phytochemicals were analyzed from sprouts of two melon Cultivars (SV9424ML and Thales) subjected to different Salt treatments during sprouting (0_mM, 12.5_mM, 25_mM, and 50 mM).

Melon sprouts were analyzed for their content in main phenolic compound groups (see Section 2.5), and the results are depicted in Figure 2 and Figure 3. In our study, significant results were obtained for all the variables of interest due to the effects of Cultivar, Salt, and Harvest, along with their interactions.

Melon Cultivar significantly affected the total phenolic compound content in sprouts, with Thales exhibiting much higher values (significant at *p* < 0.01) compared to SV9424ML (on average, 51.64 vs. 30.76 mg kg^−1^ DW, respectively) regardless of Salts and Harvests (Figure 2A). In particular, Thales sprouts were characterized by higher levels of total flavonoids (+50.7% compared to SV9424ML, averaged across Salts and Harvests), hydroxycinnamic acids (on average, +79.7% compared to SV9424ML), and hydroxybenzoic acids (+269.0% compared to SV9424ML, averaged across Salts and Harvests) (Figure 2B, Figure 2C, and Figure 2D, respectively); however, Harvest influenced the abundance of specific phenolic compound classes.

Figure 3A–C reports the variations percentage of the total phenolic compound content of ready-to-eat sprouts at 2 WAS compared to 1 WAS, allowing for a quick assessment of the impact of harvest timing, which is the result of different germination speeds, on the phytochemical composition of sprouts. Indeed, sprouts harvested at 2 WAS had lower contents of total flavonoids (−31.3% averaged across Cultivars and Salts) and hydroxybenzoic acids (−73.0% averaged across Cultivars and Salts), as well as considerably higher content of hydroxycinnamic acids (+298.6% averaged across Cultivars and Salts), regardless of the melon Cultivar (Figure 3B and Figure 3C, respectively). Nevertheless, the magnitude of observed trends depended on both Cultivars and Salts, with the most marked variations—either positive or negative—recorded for Thales, regardless of Salts (−81.2%, +427.4%, and −48.9% for hydroxybenzoic acids, hydroxycinnamic acids, and flavonoids, respectively) and at 25_mM, regardless of Cultivars (−76.2%, +443.7%, and −34.8% for hydroxybenzoic acids, hydroxycinnamic acids, and flavonoids, respectively) (Figure 3A–C). These effects allowed for an identifiable trend in terms of total phenolic compounds: SV9424ML sprouts at 2 WAS showed higher (+14.0% on average) total phenolic compound values compared to 1 WAS sprouts, particularly at 25_mM and 50_mM (+20% and +22%, respectively); in Thales all salt treatments induced a reduction in total phenolic compound content in ready-to-eat sprouts at 2 WAS, with significant differences (at *p* < 0.01) observed at 50_mM (−20.3%) (Figure 2A; see also Figure 3A).

We also observed a clear effect of the Cultivar × Salt interaction. In particular, for Thales, we registered a +14.8% increase in the total phenolic compounds content up to 25_mM compared to 0_mM (averaged over Harvests) (Figure 2A). The effects of Salts on individual classes of phenolic compounds were more noticeable when their content in the sprouts was higher—being related to Harvests and germination speed. Specifically, the effects were evident in sprouts harvested at 1 WAS for flavonoids and hydroxybenzoic acids (Figure 2B and Figure 2D, respectively), and at 2 WAS for hydroxycinnamic acids (Figure 2C). At 1 WAS, Salt significantly induced total flavonoid accumulation in sprout tissues, with the highest value recorded at 25_mM (45.23 mg kg^−1^ DW) (Figure 2B) as well as higher accumulation of total hydroxybenzoic acids (5.48 vs. 9.12 mg kg^−1^ DW at 0_mM and 25_mM, respectively) (Figure 2C). Also, 25_mM Salt allowed a significantly higher content of hydroxycinnamic acids in Thales sprouts harvested at 2 WAS (35.73 mg kg^−1^ DW) (Figure 2D). On the other hand, no significant differences in the total phenolic compound content were observed for SV9424ML in response to Salt (Figure 2A), in terms of hydroxycinnamic and hydroxybenzoic acids, independently of Harvests (Figure 2C and Figure 2D, respectively). Slight variations were recorded only in the total flavonoid accumulation on SV942ML sprouts, and the highest Salt concentrations (i.e., 25_mM and 50_mM) appeared to significantly reduce this parameter at 1 WAS (Figure 2B).

#### 3.2.2. Single Phenolic Compounds

Delving into the effects of Cultivar, Salt, and Harvest on individual phenolic compounds (both phenolic acids and flavonoids) is extremely complex. Generally, the observed differences among the phenolics classes were attributable to changes in the most representative compounds of each class, in response to experimental treatments, although specific responses should still be considered.

Flavonoids represented on average 61.9% of the total phenolic compounds. Regardless of Cultivar, Ori was the predominant flavone, and its changes effectively contributed to explaining the differences observed between ready-to-eat sprouts at 1 WAS and 2 WAS (on average, −33.6% for SV9424ML and −52.5% for Thales at 2 WAS, compared to 1 WAS), but not those related to Salt (Table 2). Indeed, under moderate salt concentration (i.e., 25_mM) Thales sprouts at 1 WAS accumulated luteolin and diosmetin (+71.3% and +89.0% compared to 0_mM, respectively). Interestingly, and in contrast to Thales, luteolin and diosmetin contents increased significantly in SV9424ML sprouts harvested at 2 WAS (Table 2).

In general, we observed low levels of hydroxybenzoic acids in melon sprouts. Vanillic acid is the most accumulated compound with values ranging from 0.433 mg kg^−1^ DW (SV9424ML sprouts obtained at 0_mM and harvested at 2 WAS) to 5.132 mg kg^−1^ DW (Thales sprouts obtained at 25_mM and harvested at 1 WAS) (Table 3).

Hydroxycinnamic acids consisted of 82.1% of the total phenolic acids investigated in melon sprouts. Among these, the significant increase in p-coumaric acid values (69.4% of total hydroxycinnamic acids) in ready-to-eat sprouts at 2 WAS, followed by the significant enhancement of ferulic acid content (28.1% of total hydroxycinnamic acids), mostly contributed to explaining the observed variations induced by Harvest (Table 4). Moreover at 2 WAS, moderate salinity (i.e., 25_mM treatment) elicited *p*-coumaric acid and ferulic acid accumulations in a Cultivar-dependent manner: in Thales sprouts, salinity boosted the content of *p*-coumaric acid, while in SV9424ML it mainly boosted the content of ferulic acid (Table 4).

#### 3.2.3. Principal Component Analysis

**Figure 4:**
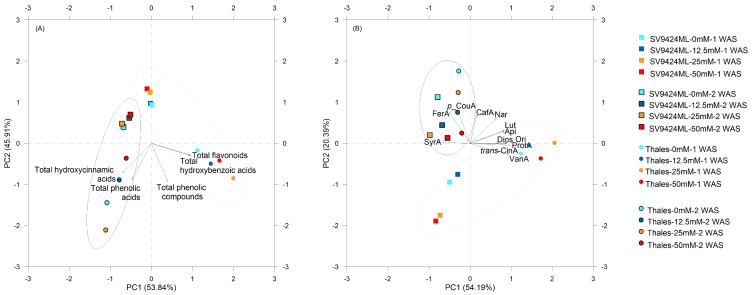
Two-dimensional correlation biplot from principal component analysis (PCA) performed on data observed in sprouts obtained from seeds of two melon Cultivars (SV9424ML and Thales) harvested at two different dates [at 1 and 2 weeks after sowing (WAS); Harvest] and subjected to different Salt treatments during sprouting (0_mM, 12.5_mM, 25_mM, and 50 mM). For both (**A**) and (**B**), symbols show the standardized scores on PC1 (x-axis) and PC2 (y-axis) for the sixteen treatments (linear combination of Cultivar × Harvest × Salt; see also the legend in the graph). Vectors’ coordinates represent the correlations between standardized variables and PCs: (**A**) classes of phenolic compounds and (**B**) single detected phenolic compounds. The total hydroxybenzoic acids include protocatechuic acid (ProtA), vanillic acid (VanA), and syringic acid (SyrA); the total hydroxycinnamic acids include caffeic acid (CafA), *p*-coumaric acid (*p*-CouA), ferulic acid (FerA), *trans*-cinnamic acid (*trans*-CinA); the total flavonoids include apigenin (Api), luteolin (Lut), diosmetin (Dios), orientin (Ori), naringenin (Nar); the total phenolic acids and the total phenolic compounds refer to the sum of the investigated molecular classes. Gray circles highlight sprouts harvested at 1 WAS, while black circles denote sprouts harvested at 2 WAS. The first two principal components explained 99.8% and 74.6% of the total variation for PCAtotal and PCAsingle, respectively. The resulting correlation biplots of the first and the second principal components (PC1 and PC2) are shown in (**A**) (PCAtotal) and (**B**) (PCAsingle).

Two distinct PCAs were performed on a 5 × 5 matrix (from the analyzed parameters, as total phenolic compounds, total flavonoids, total hydroxycinnamic acids, total hydroxybenzoic acids, and total phenolic acids; PCAtotal) and a 12 × 12 matrix (from the single investigated phenolic compounds; PCAsingle) for all 16 treatments (linear combination of 2 Cultivar, 4 Salt, and 2 Harvest) listed in Figure 4.

Focusing on PCAtotal, PC1 clustered Harvests, with 2 WAS clearly positioned on the left half (highlighted also by the black circle); treatments (e.g., Cultivar) were also clearly discriminated by PC2, with Thales positioned in the lower half and SV9424ML in the upper half. PCs did not separate Salt treatments, despite some clear tendencies emerging for Thales (Figure 4A). Considering factor loadings, higher content of total flavonoids (PC1: 0.96) and total hydroxybenzoic acids (PC1: 0.95) were associated with treatments showing higher positive scores on PC1 (in particular, Thales-25mM-1WAS: 1.99). On the other hand, ready-to-eat sprouts at 2 WAS were mainly described by high values of total hydroxycinnamic acids (especially for Thales), which increased in a Harvest-dependent manner. Moreover, PC2 highlighted the Cultivar effect on the phytochemical composition of melon sprouts, confirming the higher observed values in Thales (Figure 4A).

In PCAsingle, PCs (mainly PC2) clustered significantly by Harvest (1 WAS: gray circle; 2 WAS: dark circle), but the other effects were more complex to interpret. Higher factor loadings on PC1 were recorded for protocatechuic acid, vanillic acid, orientin, and diosmetin (0.98, 0.94, 0.89, and 0.89, respectively), which were related to higher content in Thales-25mM-1WAS (Figure 4B). Moreover, Thales-25mM-2WAS was characterized by higher factor scores on PC2 (1.24) related to higher *p*-coumaric acid content (Figure 4B).

## 4. Discussion

This study explored the potential of using melon seeds—typically a by-product of fruit deseeding—as raw material for sprout production [26,27]. In addition, the application of salinity as an abiotic elicitor was investigated as a strategy to boost the phytochemical content, offering a sustainable approach to the production of high-value plant-based foods [28,29].

The initial observation was the significant intrinsic variability within the same seed batch, which notably influenced the harvest performances of sprouts, regardless of Salt in the sprouting media. We evaluated two distinct Harvests separated by approximately one week; in this scenario, the effect was primarily attributable to germination speed. However, it should be noted that for each Harvest, Salt (i.e., 25_mM and 50_mM) delayed harvests by 1–2 days compared to 0_mM (see Materials and Methods section), confirming the effects of salinity on early growth performance [23]. In fact, seed germination faces some problems upon exposure to salinity, as at first, imbibition is affected by the lower solute potential of the soil solution. Salt and water stress might then modify the enzyme activities, exceed the ions uptake and reduce the mobility of inorganic nutrients to developing tissues, disturb nitrogen metabolism, and reduce the hydrolysis and the utilization of food reserves [30].

It is also noteworthy that the sprouts were harvested when they reached the ready-to-eat stage. The percentages of ready-to-eat sprouts at 1 WAS or 2 WAS were principally influenced by genotype. Surprisingly, salt had only a slight effect on the shift in sprout production timing (i.e., the increase in the percentage of ready-to-eat sprouts at 2WAS) and the level of salt concentration which caused this delay varied in a genotype-dependent manner (greater effect at 50_mM for Thales and at 12.5_mM for SV9424ML). Indeed, salt generally tends to sharply increase the average germination (and/or emergence) timing in other cucurbits crops [23], including melon [31].

In any case, addressing staggered germination—for example by assessing seed vigor and separating seed lots based on germination speed and/or by using some priming techniques—is crucial given the short cycle required to obtain sprouts, which limits opportunities for standardization. It poses challenges for both edible sprout production and phytochemical extraction, due to noticeable quantitative and qualitative variations in sprout growth and chemical composition across Harvests. Indeed, at 2 WAS—despite Cultivar-related differences—we observed a general sharp reduction in the SL/RL ratio. Specifically, the sprouts were characterized by higher rootlet lengths, as well as a strong modification in the phenolic composition of the young tissues, i.e., lower flavonoids and hydroxybenzoic acids and higher hydroxycinnamic acids compared to 1 WAS. The causes remain to be fully investigated and could be due to the up-or down-regulation of key enzymes in the biosynthetic pathways, or to the enzymatic degradation rates of the phenolic compounds [32]. These changes may be a response to the prolonged germination phase encountered at 2 WAS, which could have been induced by specific stressful conditions for sprouts.

Additionally, the salinity levels resulted in further and potentially stressful conditions, inducing the activation of various physiological and biochemical mechanisms to cope with stress, including, among others, changes in morphology as well as biochemical adaptations—i.e., antioxidative metabolism responses [33,34].

Firstly, regardless of Cultivars, we observed a significant increase in sprout FW at moderate salt levels—25_mM or 12.5_mM varying with Harvests—indicating that, to a certain extent, salinity may positively impact yields, as already observed in broccoli sprouts and microgreens [35,36]. With the moderate salinity we imposed, it is likely that some osmotic adjustment occurred, which contributed to maintaining high cellular turgor, cellular expansion, and growth [37]. However, the increase in FWs did not correspond to an overall increase in dry biomass, suggesting that the observed differences are likely to be due to the decrease in DM under the same salinity treatments [36]. Interestingly, at moderate salinity (25_mM), Thales showed a clear distribution of the biomass towards roots, as highlighted by the increased root length, resulting in a significant decrease in the shoot-to-root ratio. This shift in SL/RL ratio is a common mechanism of plant resistance/survival under saline conditions, primarily associated with factors related to water stress (osmotic effects) rather than to specific effects of salt [33,38]. Moreover, the elongation of the main root is crucial for seedling establishment and represents a prominent feature used to identify salt stress-related mechanisms [39]. It is worth specifying that, in our study, this response appears closely tied to genotype-specific tolerance and adaptation mechanisms, which were not observed in SV9424ML. In any case, and regardless of Cultivars, higher salinity levels (50_mM) corresponded to significant constraints on sprout elongation, as previously observed in sprouts of other species [16,37,38].

Secondly, Salt induced significant and genotype-dependent changes in the phytochemical content of melon sprouts, both between Harvests and within the same Harvest, despite the observed differences being milder than expected from the literature available for other species [6]. Indeed, salinity generally triggers oxidative stress in plants, prompting early synthesis of antioxidants like phenolic compounds to counteract its damaging effects [30,40].

In particular, salinity contributed to emphasizing the observed variations in phenolic composition among ready-to-eat sprouts at 1 WAS and 2 WAS, i.e., the difference between Harvests. In Thales, unlike at 0_mM, all salt levels seemed to reduce the total phenolic compounds in melon ready-to-eat sprouts at 2 WAS compared to 1 WAS. Moreover, 25_mM of salt solutions as sprouting media exacerbated positive variations in hydroxycinnamic acids at 2 WAS. Based on the literature on tomato exposed to salt stress, we might speculate that this could be attributed to the increased activity of the key limiting enzyme cinnamate 3-hydroxylase involved in the regulation of the hydroxycinnamic acids pathway [32]. In contrast, in SV9424ML, the contribution of salt to variations between ready-to-eat sprouts at 1 WAS or 2 WAS not as pronounced as in Thales, despite a clear trend showing higher phenolic compound levels in 2 WAS sprouts. This result is not surprising, considering that the phytochemical content accumulation during sprouting, as well as the non-enzymatic mechanisms of stress responses, vary with genotype (see, for example, Kruthika and Jithesh [41] in cereal seedlings; Irik and Bikmaz [23] in *Cucurbita pepo*).

At the same time, salinity-induced variation in phenolic compounds—total and single investigated classes of phenolic compounds—within the same Harvest time occurred only in Thales, and as long as observed in sprout growth, moderate salt levels (25_mM) consistently resulted in higher values, confirming previously results [42,43].

In describing the effects of Cultivars, Harvests, and Salts, we primarily focused on total phenolic compounds to provide a general overview, as our analysis involved the sum of individual phenolic compounds. However, the variation in single phenolic compounds could offer highly valuable insights, especially on specific responses induced by salinity in melon sprouts, although the interaction between factors is even more complex for individual phenolic compounds. Moreover, given the pioneering nature of this study, a comparison with the existing literature is nearly impossible. Anyway, the single compounds identified both within phenolic acids and flavonoids were detected in seeds, and often in other parts of melon fruits [8,44]. Differences with respect to our findings might be attributable to different melon cultivars, as well as extraction and analytical methods.

Among the most interesting findings, it was observed that moderate salt levels influenced the accumulation of lutein and diosmetin among flavonoids, and of *p*-coumaric and ferulic acid among hydroxycinnamic acids, with differences across Harvests and Cultivars. In particular, Lut accumulation—which was previously reported under mild salt stress in *Lonicera japonica*, a perennial twining woody vine used in traditional Chinese and Japanese medicine [45]—varied with Harvests, being induced by 25_mM solutions in Thales ready-to-eat sprouts at 1 WAS and by 12.5_mM salt level in SV9424ML ready-to-eat sprouts at 2 WAS. Moreover, the significant accumulation of *p*-coumaric in Thales at 2 WAS confirmed the potential role of this phenolic compound in cell wall remodeling and elongation under salt stress, despite altered turgor pressure [18]. It should also be noted that *p*-coumaric has garnered significant attention in the literature, with research indicating its potential as a promising strategy to mitigate salt stress [46]. Conversely, ferulic acid was accumulated in response to mild salinity in SV9424ML ready-to-eat sprouts at 2 WAS, as previously indicated in some *Triticum* genotypes (see literature within [6]). Moreover, hydroxycinnamic acids are often reported to be more effective antioxidants than hydroxybenzoic ones [47], and for this reason the way for their production in the shikimate pathway might be preferably induced, which would explain their increase under salinity. By contrast, the decrease observed in hydroxybenzoic acid might be due to their involvement in other pathways to counteract the oxidative stress caused by salinity. For example, lignin biosynthesis plays an important role in plant adaptation against salinity and would involve the production of hydroxycinnamic acids (e.g., *p*-coumaric, ferulic acids) for monolignols formation, and the consumption of hydroxybenzoic acids (e.g., vanillic and syringic acids) as lignin constituents [47].

In this study, PCA effectively elucidated and synthesized the complex relationships between experimental treatments and the accumulation of phenolic compounds in melon sprouts, allowing for broader conclusions to be drawn. Firstly, PCAtotal effectively separated treatments based on harvest time and Cultivar. The clustering of 2WAS treatments along the PC1 correlated with increased levels of total hydroxycinnamic acids, highlighting the influence of germination speed on the accumulation of different phenolic compound groups. PC2 further differentiated between Cultivars, demonstrating higher phytochemical accumulation in Thales compared to SV9424ML, thereby indicating potential for cultivar-specific optimization in sprout production derived from by-products.

In the more detailed PCAsingle, PCA successfully identified individual PhCs contributing to the observed trends, also reflecting the patterns observed in PCAtotal. Interestingly, the accumulation of individual phenolic compounds like protocatechuic acid, vanillic acid, orientin, and diosmetin appeared to correlate with the response of Thales to salt stress.

Lastly, the limited ability of PCA to differentiate salt treatments suggested and confirmed that the accumulation of phytochemicals in melon sprouts was more strongly influenced by other investigated factors, e.g., harvest time—as a result of germination speed—and Cultivar.

## 5. Conclusions

Despite the complexity of the results, several important insights can be gathered from our study.

Primarily, within melon sprouts sourced from waste seeds, the variability in germination speed due to intra-lot seed differences significantly influenced the time to reach the ready-to-eat stage, as well as sprout growth and chemical composition. Ready-to-eat sprouts at two weeks after sowing showed higher levels of hydroxycinnamic acids, while those harvested at one week had elevated flavonoids and hydroxybenzoic acids content, although no substantial overall variation in the content of total phenolic compounds was observed.

Secondly, salt elicited the accumulation of phenolic compounds in melon sprouts in a cultivar-dependent manner, with Thales exhibiting greater adaptation to salinity (up to 25_mM), driven by both sprout growth and biochemical mechanisms.

This may be linked to the physiological impact of salinity during germination, which could reduce water uptake and alter enzyme activities. Under stress, the increase in hydroxycinnamic acids could reflect their more effective antioxidant role and their involvement in lignin biosynthesis, crucial for structural reinforcement.

Our results suggest that the sprouting of by-product seeds of vegetables should be finely tuned, considering the intra-lot seed variability, to maximize either sprout biomass or phytochemical yield, thus addressing—depending on genotype and sprouting conditions—the needs of fresh consumption and/or industrial extraction.

## Figures and Tables

**Table 1:** Root length (RL, mm sprout^−1^), shoot length (SL, mm sprout^−1^), SL to RL ratios (SL/RL), fresh weight (FW, mg sprout^−1^), and dry matter (DM, %) as observed in sprouts obtained from seeds of two melon Cultivars (SV9424ML and Thales) subjected to different Salt treatments during sprouting (0_mM, 12.5_mM, 25_mM, and 50 mM). Sprouts were harvested (Harvest) at 1 and 2 weeks after sowing (WAS). Three-way ANOVA was applied considering Cultivar as the first, Salt as the second, and Harvest as the third factor. Data represent the means of *n* = 4 independent replicates; when ANOVA detected significant differences, means separation was performed using Fisher’s least significant difference (LSD) test at *p* < 0.05.

Effects	RL (mm sprout^−1^)			SL (mm sprout^−1^)			SL/RL			FW (mg sprout^−1^)			DM (%)		
SV9424ML	1 WAS	2 WAS	Mean	1 WAS	2 WAS	Mean	1 WAS	2 WAS	Mean	1 WAS	2 WAS	Mean	1 WAS	2 WAS	Mean
0_mM	90.3	109.7	100.0	68.7	23.1	45.9	0.762	0.215	0.489	201.3	186.2	193.8	6.56	5.83	6.20
12.5_mM	95.0	95.3	95.2	68.2	26.3	47.3	0.717	0.275	0.496	194.4	243.1	218.8	6.26	4.96	5.61
25_mM	84.0	77.5	80.8	63.2	23.0	43.1	0.749	0.303	0.526	232.7	195.5	214.1	5.99	5.20	5.59
50_mM	74.8	91.3	83.1	39.0	23.8	31.4	0.519	0.261	0.390	201.0	205.9	203.5	7.00	5.82	6.41
Mean	86.0	93.5	89.8	59.8	24.1	41.9	0.687	0.264	0.475	207.4	207.7	207.5	6.45	5.45	5.95
Thales															
0_mM	101.3	126.1	113.7	64.9	28.2	46.5	0.648	0.230	0.439	240.4	180.3	210.4	6.75	6.80	6.78
12.5_mM	89.3	114.0	101.7	69.8	28.3	49.1	0.817	0.264	0.541	229.4	276.8	253.1	6.63	5.48	6.05
25_mM	130.5	124.3	127.4	62.8	26.0	44.4	0.482	0.209	0.346	266.8	199.7	233.3	5.96	6.51	6.24
50_mM	94.7	105.3	100.0	50.2	27.8	39.0	0.530	0.275	0.402	237.6	233.4	235.5	6.66	6.17	6.41
Mean	104.0	117.5	110.7	61.9	27.6	44.8	0.619	0.245	0.432	243.5	222.5	233.0	6.50	6.24	6.37
F-test															
Cultivar	** (7.44)			* (1.08)			* (0.0171)			** (7.51)			** (0.089)		
Salt	* (10.52)			** (1.52)			** (0.0242)			* (10.63)			** (0.127)		
Harvest	** (7.44)			** (1.08)			** (0.0171)			n.s.			** (0.089)		
Cultivar × Salt	** (14.88)			n.s.			** (0.0342)			n.s.			n.s.		
Cultivar × Harvest	n.s.			n.s.			n.s.			n.s.			** (0.127)		
Salt × Harvest	n.s.			** (2.15)			** (0.0342)			** (15.03)			** (0.179)		
Cultivar× Salt × Harvest	n.s.			n.s.			* (0.0484)			n.s.			n.s.		
LSD															
Cultivar	9.92			2.16			0.0344			15.11			0.180		
Salt	14.04			3.06			0.0486			21.36			0.254		
Harvest	9.92			2.16			0.0344			--			0.180		
Cultivar × Salt	19.85			--			0.0688			--			--		
Cultivar × Harvest	--			--			0.0486			--			0.254		
Salt × Harvest	--			4.33			0.0688			30.21			0.360		
Cultivar × Salt × Harvest	--			--			0.0973			--			--		

Three-way ANOVA: n.s. not significant; * *p* < 0.05; ** *p* < 0.01. Degrees of freedoms: Cultivar, 1; Salt, 3; Harvest, 1; Cultivar × Salt, 3; Cultivar × Harvest, 1; Cultivar × Salt × Harvest, 3; residues, 48. In brackets: standard errors of the differences between means (s.e.d.).

**Table 2:** Flavonoids (mg kg^−1^ dry weight, DW)—(i) flavones (apigenin; luteolin; diosmetin; orientin) and (ii) flavanones (naringenin)—as observed in sprouts obtained from seeds of two melon Cultivars (SV9424ML and Thales) subjected to different Salt treatments during sprouting (0_mM, 12.5_mM, 25_mM, and 50 mM). Sprouts were harvested (Harvest) at 1 and 2 weeks after sowing (WAS). Three-way ANOVA was applied considering Cultivar as the first, Salt as the second, and Harvest as the third factor. Data represent the means of *n* = 4 independent replicates; when ANOVA detected significant differences, means separation was performed using Fisher’s least significant difference (LSD) test at *p* < 0.05.

Effects	Flavones (mg kg^−1^ DW)												Flavanones (mg kg^−1^ DW)		
	Apigenin			Luteolin			Diosmetin			Orientin			Naringenin		
SV9424ML	1 WAS	2 WAS	Mean	1 WAS	2 WAS	Mean	1 WAS	2 WAS	Mean	1 WAS	2 WAS	Mean	1 WAS	2 WAS	Mean
0_mM	0.045	0.049	0.047	0.566	2.005	1.286	2.57	3.47	3.02	20.26	12.39	16.33	0.259	0.337	0.298
12.5_mM	0.062	0.058	0.060	0.531	2.480	1.506	2.58	4.55	3.56	18.99	12.17	15.58	0.323	0.291	0.307
25_mM	0.038	0.049	0.043	0.525	1.603	1.064	2.68	3.53	3.10	17.45	12.04	14.75	0.173	0.210	0.191
50_mM	0.034	0.060	0.047	0.528	2.020	1.274	2.41	4.49	3.45	17.66	12.80	15.23	0.152	0.306	0.229
Mean	0.045	0.054	0.049	0.538	2.027	1.282	2.56	4.01	3.28	18.59	12.35	15.47	0.227	0.286	0.256
Thales															
0_mM	0.068	0.053	0.061	2.614	1.547	2.081	6.07	4.63	5.35	27.97	13.00	20.48	0.382	0.322	0.352
12.5_mM	0.063	0.056	0.060	3.332	2.033	2.683	6.71	5.70	6.21	28.99	13.01	21.00	0.379	0.349	0.364
25_mM	0.078	0.055	0.066	4.476	1.742	3.109	11.47	5.14	8.31	28.85	13.85	21.35	0.354	0.357	0.356
50_mM	0.074	0.055	0.064	4.092	1.717	2.904	9.10	5.08	7.09	27.02	13.69	20.35	0.363	0.326	0.344
Mean	0.071	0.055	0.063	3.628	1.760	2.694	8.34	5.14	6.74	28.21	13.39	20.80	0.370	0.338	0.354
F-test															
Cultivar	** (0.0029)			** (0.0791)			** (0.227)			** (0.325)			** (0.0177)		
Salt	n.s.			** (0.1118)			** (0.321)			n.s.			* (0.0250)		
Harvest	n.s.			* (0.0791)			** (0.227)			** (0.325)			n.s.		
Cultivar × Salt	* (0.0058)			** (0.1582)			** (0.454)			n.s.			n.s.		
Cultivar × Harvest	** (0.0041)			** (0.1118)			** (0.321)			** (0.460)			* (0.0250)		
Salt × Harvest	n.s.			** (0.1582)			** (0.454)			* (0.650)			n.s.		
Cultivar × Salt × Harvest	n.s.			* (0.2237)			** (0.643)			n.s.			n.s.		
LSD															
Cultivar	0.0058			0.1590			0.457			0.653			0.0356		
Salt	--			0.2249			0.646			--			0.0504		
Harvest	--			0.1590			0.457			0.653			--		
Cultivar × Salt	0.0117			0.3180			0.914			--			--		
Cultivar × Harvest	0.0083			0.2249			0.646			0.924			0.0504		
Salt × Harvest	--			0.3180			0.914			1.307			--		
Cultivar × Salt × Harvest	--			0.4497			1.292			--			--		

Three-way ANOVA: n.s. not significant; * *p* < 0.05; ** *p* < 0.01. Degrees of freedoms: Cultivar, 1; Salt, 3; Harvest, 1; Cultivar × Salt, 3; Cultivar × Harvest, 1; Cultivar × Salt × Harvest, 3; residues, 48. In brackets: standard errors of the differences between means (s.e.d.).

**Table 3:** Phenolic acids (mg kg^−1^ dry weight, DW)—hydroxybenzoic acids (protocatechuic acid; vanillic acid; syringic acid)—as observed in sprouts obtained from seeds of two melon Cultivars (SV9424ML and Thales) subjected to different Salt treatments during sprouting (0_mM, 12.5_mM, 25_mM, and 50 mM). Sprouts were harvested (Harvest) at 1 and 2 weeks after sowing (WAS). Three-way ANOVA was applied considering Cultivar as the first, Salt as the second, and Harvest as the third factor. Data represent the means of *n* = 4 independent replicates; when ANOVA detected significant differences, means separation was performed using Fisher’s least significant difference (LSD) test at *p* < 0.05.

Effects	Hydroxybenzoic Acids (mg kg^−1^ DW)								
	Protocatechuic Acid			Vanillic Acid			Syringic Acid		
SV9424ML	1 WAS	2 WAS	Mean	1 WAS	2 WAS	Mean	1 WAS	2 WAS	Mean
0_mM	0.010	0.003	0.006	1.546	0.433	0.990	0.076	0.126	0.101
12.5_mM	0.011	0.005	0.008	1.915	0.470	1.193	0.087	0.136	0.112
25_mM	0.017	0.004	0.010	1.996	0.487	1.241	0.084	0.122	0.103
50_mM	0.019	0.003	0.011	1.395	0.446	0.920	0.087	0.113	0.100
Mean	0.014	0.004	0.009	1.713	0.459	1.086	0.084	0.124	0.104
Thales									
0_mM	2.356	0.250	1.303	3.058	0.832	1.945	0.065	0.060	0.062
12.5_mM	2.717	0.202	1.459	4.503	1.012	2.758	0.050	0.065	0.058
25_mM	3.941	0.246	2.093	5.132	1.286	3.209	0.050	0.069	0.059
50_mM	3.436	0.325	1.881	4.510	1.127	2.819	0.055	0.046	0.051
Mean	3.112	0.256	1.684	4.301	1.065	2.683	0.055	0.060	0.057
F-test									
Cultivar	** (0.0734)			** (0.0681)			** (0.0045)		
Salt	** (0.1038)			** (0.0963)			n.s.		
Harvest	** (0.0734)			** (0.0681)			** (0.0045)		
Cultivar × Salt	** (0.1467)			** (0.1363)			n.s.		
Cultivar × Harvest	** (0.1038)			** (0.0963)			** (0.0064)		
Salt × Harvest	** (0.1467)			** (0.1363)			n.s.		
Cultivar × Salt × Harvest	** (0.2075)			** (0.1927)			n.s.		
LSD									
Cultivar	0.1475			0.1370			0.0091		
Salt	0.2086			0.1937			--		
Harvest	0.1475			0.1370			0.0091		
Cultivar × Salt	0.2951			0.2740			--		
Cultivar × Harvest	0.2086			0.1937			0.0129		
Salt × Harvest	0.2951			0.2740			--		
Cultivar × Salt × Harvest	0.4173			0.3874			--		

Three-way ANOVA: n.s. not significant; ** *p* < 0.01. Degrees of freedoms: Cultivar, 1; Salt, 3; Harvest, 1; Cultivar × Salt, 3; Cultivar × Harvest, 1; Cultivar × Salt × Harvest, 3; residues, 48. In brackets: standard errors of the differences between means (s.e.d.).

**Table 4:** Phenolic acids (mg kg^−1^ dry weight, DW)—hydroxycinnamic acids (caffeic acid; *p*-coumaric acid; ferulic acid; *trans*-cinnamic acid)—as observed in sprouts obtained from seeds of two melon Cultivars (SV9424ML and Thales) subjected to different Salt treatments during sprouting (0_mM, 12.5_mM, 25_mM, and 50 mM). Sprouts were harvested (Harvest) at 1 and 2 weeks after sowing (WAS). Three-way ANOVA was applied considering Cultivar as the first, Salt as the second, and Harvest as the third factor. Data represent the means of *n* = 4 independent replicates; when ANOVA detected significant differences, means separation was performed using Fisher’s least significant difference (LSD) test at *p* < 0.05.

Effects	Hydroxycinnamic Acids (mg kg^−1^ DW)											
	Caffeic Acid			*p*-Coumaric Acid			Ferulic Acid			*trans*-Cinnamic Acid		
SV9424ML	1 WAS	2 WAS	Mean	1 WAS	2 WAS	Mean	1 WAS	2 WAS	Mean	1 WAS	2 WAS	Mean
0_mM	0.076	0.125	0.100	3.50	9.32	6.41	2.42	5.31	3.86	0.209	0.148	0.179
12.5_mM	0.059	0.072	0.066	3.59	7.02	5.30	2.23	5.01	3.62	0.184	0.168	0.176
25_mM	0.045	0.063	0.054	1.99	7.23	4.61	1.94	6.96	4.45	0.219	0.261	0.240
50_mM	0.039	0.055	0.047	1.80	6.86	4.33	2.02	4.39	3.20	0.258	0.208	0.233
Mean	0.055	0.079	0.067	2.72	7.61	5.16	2.15	5.42	3.78	0.218	0.196	0.207
Thales												
0_mM	0.079	0.134	0.106	3.60	24.48	14.04	2.16	5.83	3.99	0.444	0.349	0.397
12.5_mM	0.098	0.064	0.081	3.07	20.33	11.70	2.69	4.19	3.44	0.370	0.166	0.268
25_mM	0.083	0.070	0.076	2.11	30.45	16.28	2.38	4.95	3.66	0.307	0.261	0.284
50_mM	0.062	0.059	0.060	1.89	15.89	8.89	1.86	3.61	2.73	0.329	0.221	0.275
Mean	0.080	0.082	0.081	2.67	22.79	12.73	2.27	4.64	3.46	0.363	0.249	0.306
F-test												
Cultivar	** (0.0051)			** (0.552)			n.s.			** (0.0111)		
Salt	** (0.0073)			** (0.781)			** (0.306)			** (0.0157)		
Harvest	* (0.0051)			** (0.552)			** (0.216)			** (0.0111)		
Cultivar × Salt	n.s.			** (1.104)			n.s.			** (0.0222)		
Cultivar × Harvest	* (0.0073)			** (0.781)			* (0.306)			** (0.0157)		
Salt × Harvest	** 0.0103			** (1.104)			* (0.432)			** (0.0222)		
Cultivar × Salt × Harvest	n.s.			** (1.561)			n.s.			** (0.0313)		
LSD												
Cultivar	0.0103			1.110			--			0.0223		
Salt	0.0146			1.570			0.615			0.0315		
Harvest	0.0103			1.110			0.435			0.0223		
Cultivar × Salt	--			2.220			--			0.0445		
Cultivar × Harvest	0.0146			1.570			0.615			0.0315		
Salt × Harvest	0.0207			2.220			0.869			0.0445		
Cultivar × Salt × Harvest	--			3.140			--			0.0630		

Three-way ANOVA: n.s. not significant; * *p* < 0.05; ** *p* < 0.01. Degrees of freedoms: Cultivar, 1; Salt, 3; Harvest, 1; Cultivar × Salt, 3; Cultivar × Harvest, 1; Cultivar × Salt × Harvest, 3; residues, 48. In brackets: standard errors of the differences between means (s.e.d.).

## Data Availability

The original contributions presented in the study are included in the article/Supplementary Material, further inquiries can be directed to the corresponding author.

## Supplementary Materials

The following supporting information can be downloaded at https://www.mdpi.com/article/10.3390/foods14132242/s1.
