# Supplementary figures and images for: Salt-Induced Changes in the Phenolic Content of Melon F2 Offspring Sprouts Obtained from Fruit Deseeding
Source: Foods. 2025 Jun 25;14(13):2242. doi: 10.3390/foods14132242 (PMC12248726; doi:10.3390/foods14132242)

**Figure S1.** Chromatogram of phenolic compounds detected in melon sprouts.

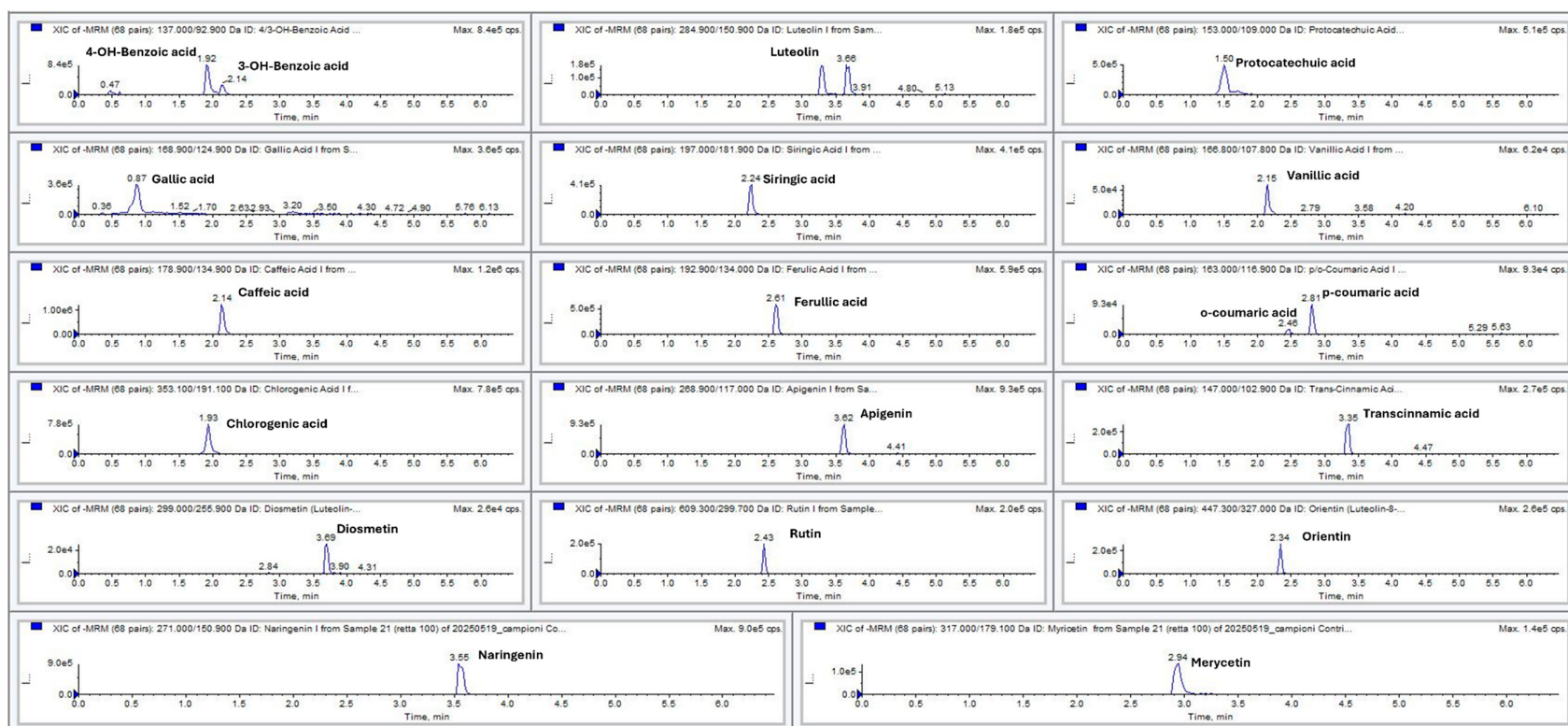

Supplement: Supplementary file 1 [file foods-14-02242-s001.zip › foods-3688466-supplementary.pdf]
